# Accurately Measuring Recombination between Closely Related HIV-1 Genomes

**DOI:** 10.1371/journal.pcbi.1000766

**Published:** 2010-04-29

**Authors:** Timothy E. Schlub, Redmond P. Smyth, Andrew J. Grimm, Johnson Mak, Miles P. Davenport

**Affiliations:** 1 Centre for Vascular Research, University of New South Wales, Sydney, New South Wales, Australia; 2 Centre for Virology, The Burnet Institute, Melbourne, Victoria, Australia; 3 Department of Biochemistry and Molecular Biology, Monash University, Melbourne, Victoria, Australia; 4 Department of Microbiology, Monash University, Melbourne, Victoria, Australia; Imperial College London, United Kingdom

## Abstract

Retroviral recombination is thought to play an important role in the generation of immune escape and multiple drug resistance by shuffling pre-existing mutations in the viral population. Current estimates of HIV-1 recombination rates are derived from measurements within reporter gene sequences or genetically divergent HIV sequences. These measurements do not mimic the recombination occurring *in vivo*, between closely related genomes. Additionally, the methods used to measure recombination make a variety of assumptions about the underlying process, and often fail to account adequately for issues such as co-infection of cells or the possibility of multiple template switches between recombination sites. We have developed a HIV-1 marker system by making a small number of codon modifications in *gag* which allow recombination to be measured over various lengths between closely related viral genomes. We have developed statistical tools to measure recombination rates that can compensate for the possibility of multiple template switches. Our results show that when multiple template switches are ignored the error is substantial, particularly when recombination rates are high, or the genomic distance is large. We demonstrate that this system is applicable to other studies to accurately measure the recombination rate and show that recombination does not occur randomly within the HIV genome.

## Author Summary

HIV's ability to generate and maintain high genetic diversity leads to multiple drug resistances and evasion from the immune system, eventually leading to immune failure and progression to AIDS. HIV maintains this diversity with a process of mutation (incorrect copying of genetic information in viral replication) and recombination (mixing two viral genomes in the creation of viral offspring). Recombination is generally studied by inserting genes encoding non-viral fluorescent proteins. However, recombination in such modified HIV genomes may not accurately reflect the level of recombination occurring within a patient infected with HIV. Additionally, recombination will go undetected in regions where the parental genomes are identical, and this effect is often ignored. We have developed a novel experimental system which allows recombination to be measured between two very closely related HIV genomes. We have also developed statistical tools to accurately calculate the recombination rate, compensating for undetectable recombination in identical regions of the parental genomes. We show that our experimental system bypasses some of the pitfalls of fluorescent recombination experiments and our tools provide a strong quantitative foundation for future studies in this area.

## Introduction

Viral diversity is one of the major obstacles to the successful eradication of HIV [1], [2]. It arises due to the interplay between mutations introduced by error-prone reverse transcription [3], high levels of viral turnover [4], retroviral recombination [5] and strong diversifying selection pressure from the immune system [2]. All retroviruses co-package two RNA genomes into each virion. Retroviral recombination occurs when the reverse transcriptase (RT) enzyme switches between co-packaged RNAs during reverse transcription (reviewed in [6], [7]). In HIV, recombination occurs much more frequently than mutation [8], and is a major determinant of viral diversification. Within infected individuals, recombination allows sequential rounds of viral escape of both antibody and T-cell recognition, resulting in loss of immune control [9], [10]. Furthermore, recombination can both promote and suppress the generation of multiple drug resistance, by creating or breaking linkages between drug resistance mutations [11]–[16]. Therefore, an accurate measurement of recombination rates directly within the HIV genome is fundamental to our understanding of HIV.

Recombination has been studied extensively, by many groups, and is typically detected by monitoring the linking of marker points from co-packaged RNA genomes into a single DNA genome. One popular method of measuring recombination is through the use of retroviral reporter systems. These systems measure recombination within a ‘foreign’ gene insert, such as genes that code for antibiotic resistance proteins, surface protein markers, and/or fluorescent proteins [8], [17]–[24]. Retroviral reporter systems have the advantage of being able to readily quantify a large number of recombination events within the gene insert. However, *in vitro* studies show that template sequence and nucleic acid structure are important determinants of the recombination process [25], [26]. Therefore, measurements of recombination rates within non-HIV ‘foreign’ gene sequences will not recapitulate recombination rates within HIV sequence. Other groups utilize the genetic variation between and within HIV subtypes, and use sequencing to monitor recombination [8], [21], [22]. These systems provide the foundation to reveal recombination events within the HIV genome. However, the use of genetically divergent RNA templates does not reflect the situation *in vivo*, where the vast majority of infected individuals are infected with a single virus which rapidly diversifies into a viral quasispecies over the course of infection [27]. The use of divergent RNA sequences can lead to confounding differences in parameters known to affect recombination, including: overall RNA homology [28], [29], RNA packaging [30], [31], and the amino acid sequence of viral proteins, such as reverse transcriptase [32]–[34]. Therefore, the recombination events detected using divergent RNA sequences most likely reflect the special case of inter-subtype recombination. Hence, there is a real need to develop a retroviral recombination system which mimics the recombination that occurs between closely related, yet genetically distinct, viruses found within an infected individual.

Recombination is detected by monitoring the linking of marker points from separate RNA genomes into a single DNA genome. Regardless of the system in which it is measured, recombination is either detected or undetected between any two marker points. This is generally interpreted as one or zero recombination events, respectively. However, with increasing genomic distance and/or recombination rate, there is an increased likelihood that there will be multiple template switches between any two marker points which go undetected. Consequently, with high rates of recombination and/or genomic distances between marker points, there is a greater chance of underestimating recombination rates due to multiple template switches. These possibilities have been mentioned previously [20], [24], [35], [36]. However, there is no current standard method to calculate recombination rates over multiple genetic regions of varying lengths that also compensates for the possibility of multiple template switches between marker points. Additionally there exists no theoretical estimate for the error when recombination is measured without compensating for multiple template switches, as is often the case.

Here, we present a novel experimental method based on limited codon modification of the HIV genome which does not change the infectivity of the virus or any viral protein. This allows the measurement of recombination between closely related genomes analogous to those found in the quasispecies of an infected individual. This system measures recombination in different gene segments, allowing the identification of possible recombination ‘hotspots’, where template switches occur at higher frequencies. We then develop statistical tools to calculate an ‘optimal recombination rate’ that reproduces observed recombination frequencies, taking into account multiple template switches. These tools demonstrate the error in calculating crude recombination rates (that do not consider multiple template switches) and emphasize the necessity for careful data analysis. These tools also provide the basis to quantify statistical differences in recombination rates in various regions of the HIV genome, under different conditions, or infection with different target cells. Finally, our analysis allows for testing and subsequent validation of some inherent assumptions and sources of error in the experimental design. We compare our analytic procedure with previously published studies and find that our approach avoids some of the potential pitfalls of using reporter gene inserts.

## Results

### Modeling the effects of multiple template switches on the observable rate of recombination

**Figure 1:**
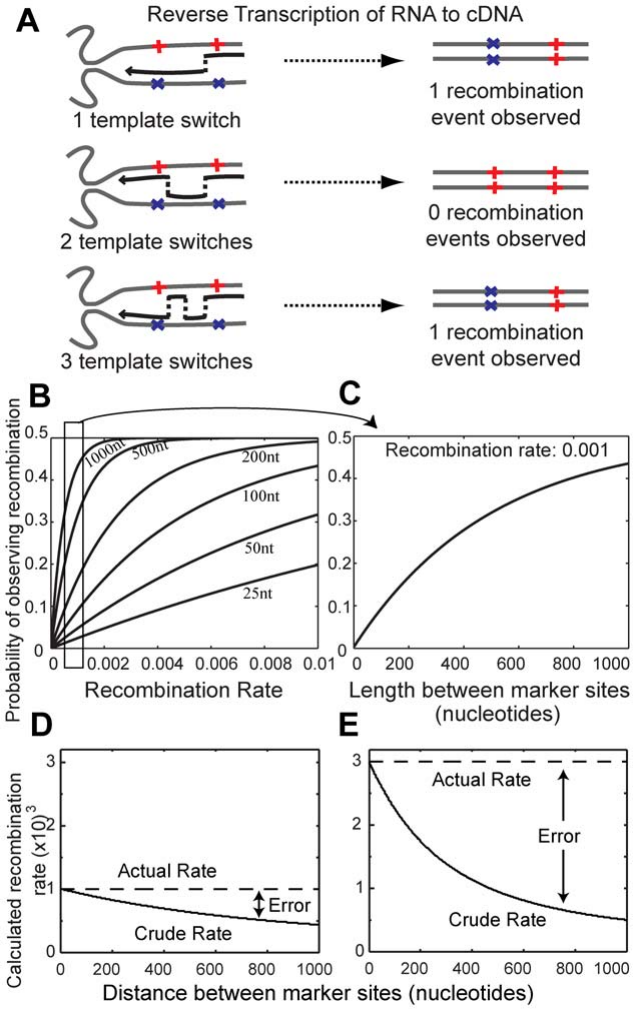
Multiple template switches change the observable recombination rate. (A) Recombination occurs during reverse transcription when RT switches from one co-packaged RNA template to another. Marker sites in one RNA template allow the detection of recombination. However, the exact number of template switches cannot be known. That is, recombination is only observed with any odd number of template switches and recombination is not observed with zero, or any even number of template switches. (B) The probability of observing a recombination crossover changes with recombination rate (measured as “recombination events per nucleotide per round of infection [REPN]) and the length over which recombination is observed. For each length (length in nucleotides of RNA shown below each line), each recombination rate produces a unique probability of observing a recombination. (C) Profiles a snapshot of the probability of observing a recombination over different lengths with a constant recombination rate 0.001 REPN. (D and E) Using a crude formula for calculating the recombination rate (*r = c/nl*, where *c* is the number of template switches detected, *n* is the number of sequences, and *l* is the distance between marker sites) that does not take into account multiple template switches underestimates the actual rate. This error increases with genomic distance and recombination rate. The probability of observing recombination is calculated with the Poisson approximation derived in Materials and Methods (Equation A).

Recombination is measured by analysing the cDNA that results from infection with non-identical (heterozygous) co-packaged RNA genomes. The positions in which the RNA genomes differ are called marker points. Recombination is detected only when the resulting cDNA contains a mixture of marker points from both RNA strands. It is tempting to conclude that one template switch has occurred every time recombination is detected between a set of marker points, and that no template switches occurred elsewhere. However, any even number of template switches between two fixed marker points will lead to us observing no recombination, and any odd number will result in us observing a single recombination event (Figure 1A). An important consequence of this fact is that the probability of observing a recombination event is a function of the genomic distance between the markers and the overall recombination rate. We created a model of recombination which takes into account the possibility of not detecting recombination events (see Materials and Methods). Our recombination rate calculation (denoted ‘optimal’ recombination rate) reveals the relationship between the overall recombination rate, distance between marker points and the probability of observing a recombination event (Figures 1B and 1C). We show that for each genomic distance and overall rate of recombination, there is a unique probability of observing recombination. Furthermore, with high overall rates of recombination and large genomic distances, it becomes much more difficult to calculate the recombination rate accurately. Indeed, these probabilities eventually converge until it becomes impossible to derive the true rate of recombination because there is an equal chance of observing or not observing a recombination event.

### Ignoring multiple template switches consistently underestimates the real recombination rate

To demonstrate the consequences of ignoring multiple template switches, we utilized a simple equation (denoted ‘crude’ recombination rate calculation): *r = c/nl*, where *r* is the rate of recombination events per nucleotide per round of infection (REPN), *c* is the number of template switches detected, *n* is the number of sequences, and *l* is the genomic distance over which recombination is measured. This crude formula assumes that between marker points, at most one template switch can occur. To calculate the theoretical expected error of the ‘crude’ recombination rate we first use the ‘optimal’ recombination rate equation (Eq. A) to determine the probability of observing recombination over different genomic distances. We then use the ‘crude’ recombination rate calculation on these probabilities and find that this calculation consistently underestimates the real recombination rate. At an actual recombination rate of 0.001 REPN (lower than the median recombination rate measured in T-cells in this study), the calculated crude recombination rate is 9% lower when measured over a distance of 100 nucleotides, and 37% lower when measured over a distance of 500 nucleotides (Figure 1D). This error is even larger when the real recombination rate is 0.003, where the crude rate is 25% and 68% lower than the actual rate when measured over a distance of 100 nucleotides and 500 nucleotides respectively (Figure 1E). This error is a direct result of not considering multiple template switches, emphasizing the need for our optimal recombination rate calculation.

### Experimental detection of recombination within the HIV genome

**Figure 2:**
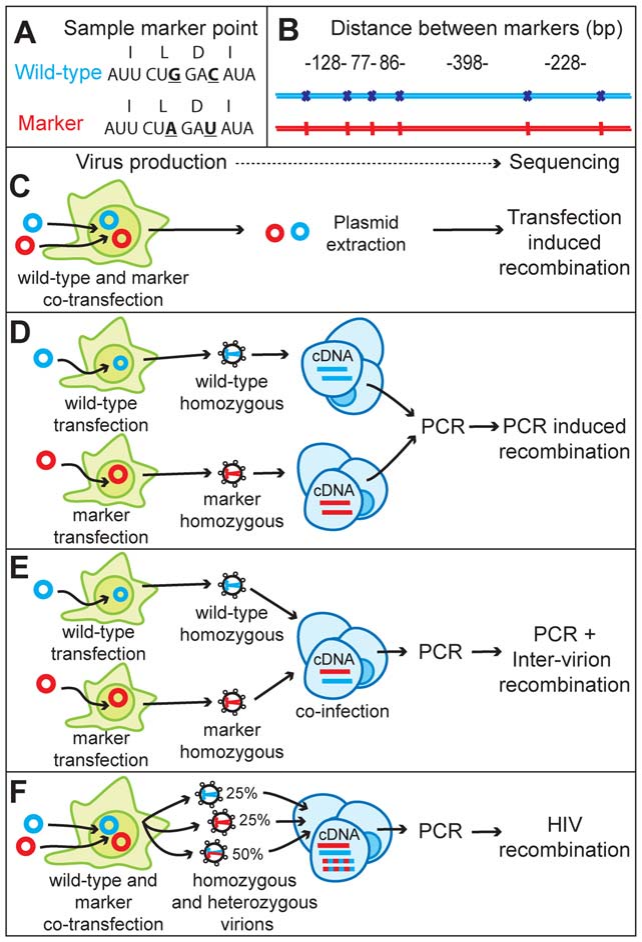
Schematic representation of the marker recombination system. (A) Marker points were introduced by genetic changes that do not alter the amino acid sequence. (B) The distances between marker points within *gag* (C) Transfection-induced recombination was measured by direct sequencing of plasmid DNA extracted from co-transfected 293T cells (D) PCR-induced recombination was measured by separately infecting T-cells with homozygous virions derived from single transfections of WT and MK plasmid. Lysates were mixed before PCR. (E) Inter-virion recombination and PCR-induced recombination was measured by infecting T-cells with homozygous virions derived from single transfections of WT and MK plasmid. (F) The recombination rate in T-cells was measured by infection of T-cells with virus produced by co-transfection of 293T cells with WT and MK plasmid. (D,E,F) Recombination rates were measured by PCR of cellular lysates, cloning and sequencing.

We sought to measure the rate of recombination directly in the HIV genome. To this end, we made a marker virus (MK) by introducing 6 codon modifications into the *gag* gene of wild-type (WT) HIV. This creates 5 regions (varying in length from 77 to 398 nucleotides) over which we can directly measure the recombination rate of a full length HIV genome (Figure 2A and 2B). These modifications neither affect the infectivity of the virus nor alter the amino acid sequence of any viral protein (Figure S1). The recombination process depends greatly on template sequence, RNA structure, the overall homology between sequences and the viral proteins involved in reverse transcription. Therefore, this system mimics the situation *in vivo*, where recombination occurs in the context of a quasispecies of highly related, yet genetically distinct viruses.

In our experimental system, a template switch observed in the DNA provirus is most likely to be the result of viral recombination during reverse transcription of the two RNA molecules co-packaged in a heterozygous virion. However, it is possible that recombination could also have occurred during a number of steps in sample preparation and sequencing. To determine the potential bias within our experimental system, we quantified experimentally-induced recombination, as follows:

Firstly, we tested the possibility of transfection-induced recombination which can occur via homologous recombination in the producer cell [37]. We measured this by direct sequencing of plasmid DNA extracted from co-transfected 293T cells (Figure 2C). Of 182 sequences of plasmid DNA extracted from transfected cells we observed zero recombination events, suggesting that this is not a source of error in our system (Table 1).

**Table 1:** Crude and optimal recombination rates in T-cells and experimental controls.

Recombination Experiment	Clones sequenced	Recombination events observed	Crude Recombination Rate (×10^−3^)	Optimal Recombination Rate (×10^−3^)	95% Confidence (Lower bound) (×10^−3^)	95% Confidence (Upper Bound) (×10^−3^)
Transfection	182	0	0	0	0	0
PCR	125	3	0.069	0.102	0	0.319
PCR+Intervirion	128	2	0.042	0.105	0	0.376
T-cells	118	32	0.814	1.451	0.773	2.738

Experiments were carried out to quantify recombination in T-cells and experimentally induced recombination. DNA from each experiment was cloned and sequenced and the number of recombination events scored. The crude and optimal recombination rate for each experimental condition was calculated. Sequence data was bootstrapped to generate 95% confidence intervals.

Secondly, we tested for PCR-induced recombination that may occur if the polymerase switches templates during PCR amplification of the viral sequences prior to sequencing. We measured this by performing two separate infections with either WT homozygous virus or MK homozygous virus (Figure 2D). In this case, recombination occurs at the usual rate between co-packaged HIV RNA strands, but template switching between these identical copies of RNA cannot be detected. These homozygous samples are mixed prior to PCR. Thus, any observable recombination can be inferred to be an artifact of the PCR. 125 sequences were obtained and 3 recombination events were detected (Table 1).

Finally, we measured the rate of ‘inter-virion’ recombination that may have occurred if the target cells were multiply infected, and retroviral recombination was occurring between the RNA molecules of different virions. To do this we co-infected cells with homozygous WT and homozygous MK virions. Thus, any intra-virion recombination would be undetected, but both inter-virion recombination and PCR-induced recombination would be detected (Figure 2E). 128 sequences were obtained and 2 recombination events were detected (Table 1).

To measure the biological rate of recombination we generated a mixture of heterozygous and homozygous virus by co-transfection. When equal amounts of two HIV plasmids are co-transfected, co-packaging of RNA into virions is random [38]. Therefore, when we co-transfected equal amounts of WT and MK plasmid, we expect 50% heterozygous virions, 25% homozygous WT virions and 25% homozygous MK virions (Figure 2F). This mix was used to infect primary T-cells. 118 sequences were obtained and 58 recombination events were detected (Table 1).

### Calculating crude and optimal recombination rates

In determining recombination rates, it is easy to assume that transfection of equal amounts of WT and MK plasmid leads to the production of 50% heterozygous and 50% homozygous virus. However, variations in the level of co-transfection will lead to the production of a different proportion of heterozygous virions than expected. This will bias the calculation of recombination rates. Our design allows us to estimate the proportion of heterozygous virions in our experiments directly from the data (as described in Materials and Methods). The estimated proportion of heterozygous virus was approximately 50% in our studies (48.6%, 45.1%, 49.7% and 46.1% for transfection, PCR, between virion and T-cell experiments respectively), indicating that there is no bias in infection rates between WT and MK virus or in the production of our heterozygous virions.

We then calculated the recombination rates for each of our experimental conditions, using both our crude and optimal recombination rate calculations (Table 1). As we detected no recombination events in our transfection-induced recombination control, the crude and optimal recombination rates were 0 REPN. From 125 and 128 sequences for the PCR-induced recombination control and the PCR-induced plus inter-virion control, we observe 3 and 2 recombination events respectively. This corresponds to an optimal recombination rate of approximately 0.1×10^−3^ REPN. For our biological sample, the crude recombination rate was calculated to be 0.81×10^−3^ REPN, and the optimal recombination rate to be 1.45×10^−3^ REPN. Thus, the crude recombination rate underestimates the optimal rate by approximately 44%. This underlines the importance of calculating recombination rates using our ‘optimal’ recombination rate calculation instead of the ‘crude’ method commonly used in the literature, which does not compensate for multiple template switches.

Using the above approach we are able to directly estimate the recombination rate from an experimental data set. However, the error of this estimate is affected by the number of sequences sampled, and their distribution. In order to determine confidence intervals for these estimates we generated probability distributions by bootstrapping the sequence data (see Materials and Methods). The 95% confidence intervals of these distributions are calculated with the Percentile Method and are shown in Table 1. Due to the high number of samples (>118 for all datasets) and relative symmetry of the bootstrap distributions (data not shown), we assume very good coverage of these confidence intervals. We conclude that the recombination rates are significantly different (at the 0.05 level) when the 95% confidence intervals do not overlap. These distributions show that the recombination rate is not significantly different between PCR induced recombination and PCR induced plus inter-virion recombination. Thus, inter-virion recombination is not a significant factor in our experimental setup. However, recombination rates were significantly different between our controls and the rate of HIV RT-induced recombination in the biological sample. The true HIV RT-induced recombination rate was then calculated with a control correction method (see Materials and Methods), that is approximately a subtraction of the two recombination rates. The RT-induced recombination rate alone is calculated to be 1.35×10^−3^ REPN in primary T-cells.

### Calculating the optimal recombination rate per nucleotide per round of infection (REPN) in fluorescent protein gene insertion experiments

Our experimental system allows recombination to be measured between closely related viral genomes. However, most recent recombination assays involve the insertion of fluorescent proteins into the HIV genome. In these systems two distinct defective genes, encoding a fluorescent protein, are inserted into different HIV genomes. A recombination event that eliminates the deactivating mutations recreates a functional fluorescent encoding gene. Recombination can then be measured via FACS analysis of infected and fluorescent protein expressing cells. This technique is capable of producing large quantitative datasets and has shown to be an effective tool to compare recombination rates under varying conditions. Generally, the extent of recombination in these systems is measured as a function of the multiplicity of infection (MOI) of fluorescent protein expressing cells and the MOI of viral infection. However these calculations are not easily comparable to calculations made for marker points separated by different genomic lengths. A clearer approach is to calculate the recombination rate in terms of ‘recombination events per nucleotide per round of infection’ (REPN), as this rate allows the prediction of the number of recombination events that will occur over any length of RNA. To demonstrate how our recombination rate calculation method can be applied to fluorescent protein studies, and to make a direct comparison of these recombination rates to our own, we analysed the data from Rhodes et al. 2005 [24].

Table 2, 3, 4, and 5 from Rhodes 2005 lists the total number of cells, infected cells, and green fluorescent protein positive (GFP+) cells when the recombination is measured over a genomic distance of 588, 300, 288 and 103 base pairs, respectively. From these ratios the GFP+ MOI/infection MOI (ratio denoted as *M*) is calculated. This ratio represents the probability of a single infection event resulting in the reconstruction of a functional GFP protein (see Materials and Methods). Recombination is only detectable from 50% of the virions (those that are heterozygous). Thus, the probability that a heterozygous infection recreates a functional GFP is *2M*. A functional GFP is only created when the two inactivating mutations are eliminated via recombination. However, the two inactivating mutations being ‘joined’ via recombination is equally likely. Thus, the probability that a heterozygous infection results in mosaic cDNA (from a nucleotide sequence perspective) is *4M*. Using equation (A) (Materials and Methods) with *R(L) = 4M* converts M into the required recombination rate measured in REPN.

**Table 2:** Calculation of optimal recombination rates from a retroviral reporter system (Rhodes et al. 2005).

	Recombination rate (×10^−3^)		
Genomic distance	Cell line 1	Cell line 2	Cell line 3
588	0.68	0.56	0.91
300	0.97	0.68	0.76
288	0.75	0.55	0.59
103	0.75	0.49	0.53

The optimal recombination rate can be calculated using equation (A) and substituting 4M for R(L), where M is the fluorescent protein MOI divided by the infection MOI (equal to the probability of a single infection event creating a fluorescent protein expressing cell). Recombination rates are similar when measured over varying genomic distances illustrating how our analytical recombination rate calculation calculates the optimal recombination rate regardless of the genomic distance over which recombination is measured.

Thus, taking into account the possibility of multiple template switches, the recombination rates for the data in Rhodes 2005 ranges from 0.49×10^−3^ to 0.97×10^−3^ (table 2). Note that the calculated optimal recombination rates in Rhodes 2005 are similar regardless of the genomic distance over which recombination is measured. This is because our analytical recombination rate calculation compensates for genomic distance when calculating the probability of multiple template switches. Our conversion of the data in Rhodes 2005 to a recombination rate per nucleotide per round of infection is in line with previous conversions by Suryavanshi and Dixit [36], who used curve fitting techniques to estimate an average recombination rate over the different lengths. The advantage of our technique is that our procedure can be applied with a standard calculator and requires no curve fitting experience or software.

### Recombination does not occur randomly in the viral genome

Crossover sites of the HIV-1 RT may consist of RNA sequence determinants that direct the RT to switch templates, and it has been suggested that RNA-RNA interactions can promote recombination *in vitro* [28], [29]. Unlike systems that measure recombination over only one region, our experimental design allows recombination to be studied in five gene segments in *gag*, which cover a total genomic distance of 917 base pairs. Our analytical recombination rate calculation allows us to calculate the optimal constant recombination rate that best describes our experimental data and compensates for the possibility of multiple template switches. This system allows us to determine: (i) if the variation in recombination along the gene is significantly different than that expected by random variation (indicating whether recombination is a random event); (ii) the optimal location for a recombination rate change (determining the marker point that separates any recombination ‘hotspots’ and ‘coldspots’); and (iii) whether a two recombination rate model better describes the observed experimental recombination data. Together, these analyses will help us to determine whether recombination occurs randomly across the viral genome.

**Figure 3:**
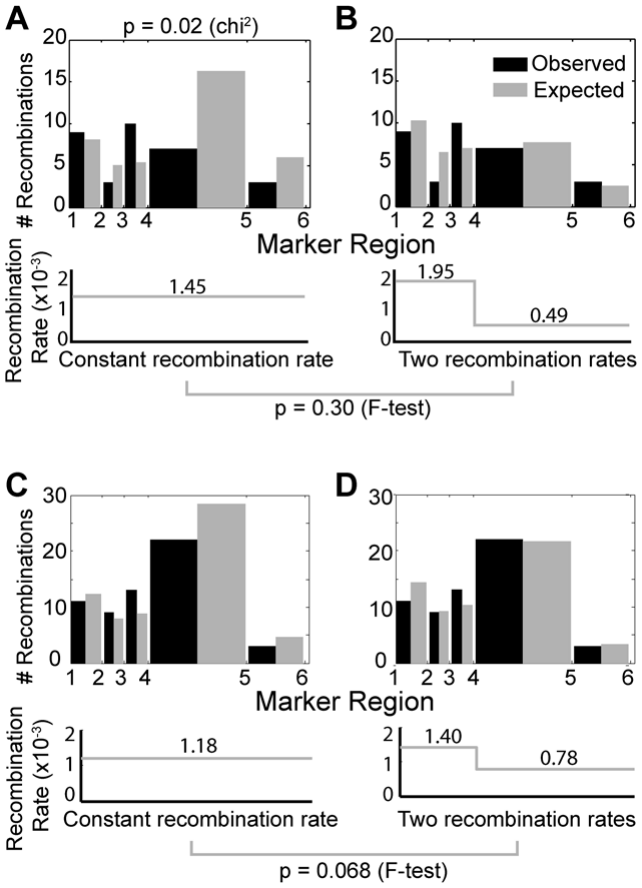
HIV recombination varies across genome. An optimal recombination rate is calculated by minimizing the chi-square value between the observed and expected frequencies of detectable template switches in each of the regions. A chi-square goodness of fit test indicates whether recombination rates are likely to vary over the entire length. (A) Variation between expected and observed frequencies (B) A dual recombination rate model was fitted and marker point 4 was the optimal location for a recombination rate switch. (C, D) In a second independent experiment we also observe that recombination is higher towards marker region 1 and lower towards marker region 6. However an F-test comparing a single recombination rate model (C) and a dual recombination rate model (D) with the switch location known from the original experiment, still did not achieve significance (p = 0.068, F-test).

We first use a chi-squared goodness of fit test to determine if the observed frequency of recombination and the expected frequency (calculated from our optimal recombination rate and compensating for multiple template switches, equation (B) Materials and Methods) are significantly different in each gene segment. Figure 3A profiles the experimentally observed and expected number of detected template switches that were recorded over the different sections of *gag*. The experimental data displays significant variation from the expected frequencies of recombination to the observed frequencies (p = 0.02) suggesting that recombination rates vary along the gene segments.

We then adjusted our mathematical model of HIV recombination to fit two optimal recombination rates along the gene segment. This was achieved by splitting the gene segment into two, and calculating each subsegments optimal recombination rate. The location of the split was optimised along marker positions 2 to 5. We find that the recombination rate is higher towards the marker site 1 end and lower towards marker site 6. The optimal location for recombination rate switch was at marker site 4 (1.95×10^−3^ and 0.49×10^−3^ REPN from sites 1–4 and 4–6 respectively) (Figure 3B). Comparing the dual recombination rate model to the original model with an F-test did not produce a significant p value (p = 0.30, Figure 3B), indicating that the dual recombination rate model did not fit significantly better to justify the additional parameters (second recombination rate and switch location). To address this further, we analyzed a second set of data and sequenced 192 cDNA strands. Again, we found that recombination is higher towards marker site 1 and lower towards marker site 6 (Figure 3C and 3D). However, an F-test comparing the one and two recombination rate models in this dataset, but this time applying the same switch location estimated in the first experiment (one less parameter in the two recombination rate model), still did not achieve significance (p = 0.068). Thus, our data support a difference in recombination rate across the gene, but was unable to identify the precise ‘hotspots’ of higher recombination.

**Figure 4:**
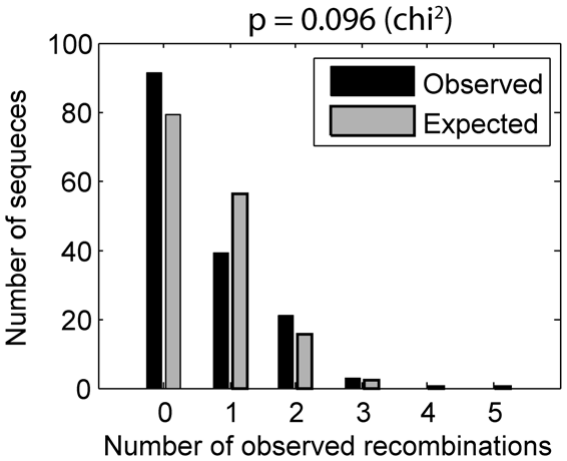
Frequencies of multiple detected template switches follows a modified Poisson distribution. The experimental assumption of an equal recombination rate amongst all sequences predicts that the frequency of multiple observed recombination events should be distributed as calculated by rate of recombination in each region, number of sequences and lengths over which recombination is measured. Large variation from the expected distribution indicates that this assumption may not be correct. Data shown is from the two heterozygous infection datasets combined (310 total sequences) corrected for control recombination (PCR).

The assumption of an equal recombination rate amongst all sequences predicts that the frequency of multiple recombination events should be Poisson distributed. However, due to the possibility of multiple template switches occurring between markers of varying genomic distances, and the possibility of varying recombination rate across the *gag* gene, the frequency of multiple detectable template switches does not follow a Poisson distribution. We calculated this distribution to compute the expected frequency of multiple detectable template switches and compare this to our experimental results (Figure 4). This calculation compensates for multiple crossovers and uses the individual recombination rate observed in each region. Our data indicates some variation from the expected frequency of multiple template switches, however this was not significant (p = 0.096).

Finally, it is possible that the limited introduction of marker points into the HIV genome altered the RNA structure in such a way as to bias the recombination process. For example, reverse transcription commencing on the MK genome may be more likely to result in recombination than reverse transcription on the WT genome due to our codon modifications. This predicts that the probability of recombination will be different when the RT is reverse transcribing a WT or MK marker point. Therefore, we compared the proportion of recombination events where recombination occurred from MK to WT, versus from WT to MK in our sequences. Of the 90 template switches observed in the pooled dataset, 42 were MK to WT and 48 were WT to MV, consistent with the null expectation of 50∶50 (p = 0.60, binomial distribution). This illustrates that our codon-modified markers have not significantly altered the RNA structure so as to bias the observed recombination rate.

## Discussion

Recombination plays an instrumental role in the evolution of HIV [39], [40] and continues to shape the global pandemic [41]. Despite the excellent progress made in understanding inter-subtype recombination [30], [42], [43], the study of recombination occurring between closely related genomes within an infected individual has been hampered by the lack of an appropriate model. Existing recombination systems are based on foreign reporter sequences, inter-subtype HIV genomes and/or intra-subtype HIV genomes with variation in amino acid sequences. Therefore, we have developed a novel marker system and associated mathematical tools that: (i) measures the recombination rate directly on the HIV genome; (ii) controls for background recombination; (iii) corrects for multiple template switching.

Our HIV recombination marker system uses genetic marker points based on the codon modification of the authentic full length HIV genome without altering the amino acid sequences. Other groups have previously measured recombination rates on the HIV genome using the divergent RNA sequences found between or within HIV subtypes or within non-viral reporter sequences. Our procedure has several advantages over previously published methods. First, we rationally introduced marker points into the HIV genome at well defined locations, by avoiding RNA sequences that are known to be important for HIV replication. These marker points allow recombination to be monitored, but do not affect the HIV replication cycle, even over multiple rounds of replication (Figure S1). This is in contrast to recombination systems using divergent RNA sequences from different viral strains, where the differential replication capacities of the virus may bias the outcome of recombination. Second, our marker system retains every virion protein and these are expressed in their correct biological context. In the case of the retroviral reporter systems, it is common to completely knockout one or more HIV proteins by replacing them with non-viral reporter protein sequence. Therefore, these reporter systems, even when attempts are made to reintroduce these proteins back into the virion, do not recapitulate the exact biological conditions occurring in the full length virus [22], [24]. Third, our silent modifications do not change the amino acid sequence of the viral proteins. This is important in light of reports that the amino acid sequence of the HIV RT affects the rate of template switching [32], [34] and that mutations in the Gag polyprotein can affect RNA packaging and recombination [44]. Therefore, it seems likely that variations in the amino acid sequence of any viral protein involved in either assembly or reverse transcription of the virus could have unintentional consequences on the rate of recombination. This would limit the utility of divergent RNA sequences, even from within the same subtype [45]. Fourth, by limiting our modifications to targeted regions of the genome, we aim to maintain overall RNA structure and homology, which are critical determinants of recombination [28], [29], [46]. We demonstrated that recombination occurred at an equal rate on our WT and MK genome; hence, our modifications do not change the rate of recombination. This indicates that the variations in the recombination rate we observe are due to differences in the RNA sequence between marker points, not to the marker points themselves.

We acknowledge that there are experimental complexities associated with the direct measurement of recombination by sequencing that can lead to the inclusion of non-viral recombination artifacts. Therefore, we carefully controlled for transfection-induced recombination, PCR-induced recombination and the effects of co-infection due to inter-virion recombination. In our study, transfection-induced recombination can be excluded as a source of error. We also show that inter-virion recombination, due to multiple infections of a cell, is not a significant source of error. By contrast, most retroviral reporter systems are biased by multiple infections. That is, in most retroviral reporter systems, multiple infections cannot be distinguished from single infections. This decreases the apparent total number of infection events, which is required to accurately calculate the recombination rate. To overcome this, these systems make use of MOI calculations which compensates for multiple infections. However, MOI calculations assume that infection events are independent and random. This is problematic in light of reports that double-infection occurs more frequently than predicted from random chance alone [47], [48], although this effect has been challenged by other data [22] and mathematical analysis [49]. Nevertheless, our system has the advantage that the recombination rate calculations are not affected by the occurrence of multiple infections. Finally, we did detect some recombination due to PCR-induced recombination but were able to optimize our PCR cycling conditions to minimize its effects. In addition, our recombination rate calculation corrects for this background to reveal the true rate of recombination. This highlights the necessity of including appropriate controls, as the effect of PCR-induced recombination has been ignored in similar studies [8], [22], [50], [51].

As recombination is measured by observing the linking of genetic marker points, all recombination systems are potentially biased by the occurrence of multiple template switches. A potential solution is to reduce the genomic distance between marker points and to evenly space them on the HIV genome. This effectively eliminates multiple template switches and any bias due to variations in genomic distance between marker points. However, it is impossible to modify the HIV genome in this way without drastically affecting the replication cycle. As a result, modifications that do not affect important RNA sequences or vary the amino acid sequences of viral proteins will always be unevenly spaced. Furthermore, increasing the frequency of marker points increases the genetic diversity between co-packaged RNAs. This is expected to decrease the observed recombination rate, as high levels of sequence identity between templates is required for efficient template switching [46], [52]. Thus, whilst reducing the genomic distance between markers can improve the ability to detect recombination, it also biases the observation by decreasing the likelihood of template switching in the first place. As multiple template switches between any two marker points occur, by definition, between identical sequences, these switches take place under optimal conditions for recombination. Therefore, a better solution is to compensate for multiple crossovers when calculating the recombination rate, as we have done.

We also calculate the theoretical estimate for the error when recombination is measured without compensating for multiple template switches and show that the width between marker points can dramatically affect the crude recombination rate estimation. For example, when the distance between marker points is 400 base pairs, an actual recombination rate of 0.001 REPN and 0.003 REPN would be crudely calculated to be 0.0007 REPN and 0.0011 REPN, respectively. This is a difference that could be interpreted as resulting from random variation alone. This effect becomes more important at higher recombination rates. This is especially significant as recombination has been reported to be 3-fold higher in macrophages than in T-cells [22], although this has been disputed by another group [18]. Regardless, we find that HIV undergoes 1.35×10^−3^ REPN in primary T-cells, which is a high rate of recombination, equivalent to 12.5 recombination events per genome every replication cycle. This is higher than when we apply our method to the data in Rhodes 2005 [24] (average recombination rate 0.69×10^−3^). However the measurements in our study are based on the HIV genome rather than non-viral reporter genes in previous studies [18], [22]. The utility of our optimal recombination rate calculation is demonstrated by the fact that the crude calculation underestimates the optimal recombination rate by 44%. In addition, our bootstrapping procedure determines the confidence intervals of the recombination rate estimate. As these confidence intervals are derived from the actual data set and take into account variable distances between markers, it enables the direct comparison of recombination rates under different experimental conditions as well as providing an additional level of accuracy to our estimation.

Our measurements on the HIV genome demonstrate that recombination does not occur randomly. Firstly, our results suggests that two or more recombination events on the same RNA strand may be observed more frequently than expected, although this was not statistically significant (p = 0.096, Figure 4). This is in line with previous work showing HIV recombination exhibiting negative interference, which is when a single recombination event increases the chance of a second recombination event taking place [8], although this is not universally agreed upon by all researchers [18]. Secondly, in two independent datasets (from different blood donors), the recombination rate appears to be lower towards marker position 6 compared to position 1 within the *gag* gene. We tested whether a dual recombination rate model fitted the data better, but this did not reach significance at the 0.05 level (p = 0.068). Therefore, the data imply that there are recombination ‘hot-’ and/or ‘cold-spots’ within the genome but this current dataset was not large enough to identify how, or precisely where, the recombination rate changes. Interestingly, comparative sequence analysis of inter-subtype recombinants also showed a reduction in recombination near the 3′ end of *gag* [43]. Although this could be due to selection, our study opens up the possibility that this region of the genome may be inherently less prone to recombination. Further studies with much larger sequence numbers will be required to determine the positions of various recombination ‘hot/coldspots’, and their respective recombination rates. The requirement of large quantities of sequencing data is a major limitation of our analytical tool. However, with the availability of next generation sequencing technology, plus the design of a marker system that has more marker points (higher level of resolution), these issues can be readily accommodated.

We have now developed appropriate statistical tools to quantify the rate of retroviral recombination taking into account the experimental procedures involved in observing recombination. We have shown how this can be used to compare recombination rates and to identify recombination hotspots within the viral genome. We also test a number of underlying biological and analytical assumptions that are often overlooked. These methods take into account the experimental and biological complexities of measuring recombination, and will provide a strong quantitative foundation for future studies in this area.

## Materials and Methods

### Ethics statement

Human primary cells were isolated from buffy packs from random (identity blocked) blood donors to the Red Cross Blood Bank. All biological samples were handled according to the Burnet Institute and the Alfred Hospital approved ethics guidelines that are in line with Australian Government regulation.

### Viruses

Homozygous virus was produced by transfection of 293T cells with either WT or MK pNL4-3. Heterozygous virus was produced by co-transfection of equal amounts of wild-type pNL4-3 and marker pNL4-3 into 293T cells. Transfections were carried out with polyethylenimine (PEI; Polysciences), and transfection efficiencies were measured using a reverse transcriptase assay [53], [54]. 36 hours post-transfection, virus containing media was harvested, clarified by centrifugation at 1,462×*g* for 30 minutes, and then passed through a 0.45µm filter to remove cellular debris. Purified virus was concentrated by ultracentrifugation at 100,000×*g* through a 20% sucrose cushion and stored at −80°C. Virus was treated with 90units/mL benzonase (Sigma) for 15 minutes at 37°C to remove contaminating plasmid DNA before use.

### Recombination assay

Stimulated PBLs were infected with equal amounts of either homozygous or heterozygous virus, as determined by a HIV-1 antigen (p24 CA) micro ELISA assay (Vironostika). Heat inactivated (2 hours at 56°C) control infections were carried out to confirm efficient removal of plasmid DNA for each sample. 6 hours post-infection 10µg/mL T-20 (Roche) was added to the cells to prevent second round replications. 24 hours post-infection cells were pelleted, lysed and full length reverse transcriptase products were quantified, as previously described [55]. A 1kb fragment of *gag* was PCR amplified using the primers (EcoRI)NL3065s [GCAgaattcGAGCTAGAACGATTCGCAG] and (BamHI)NL4066a [TATggatccTGGATTTGTTACTTGGCTCATTG] and the following conditions: initial denaturation 98°C for 30 seconds, followed by 30 rounds of cycling at 98°C for 10 seconds and 72°C for 2 minutes. PCR amplification was done in the log-linear phase as determined by real-time PCR to minimize PCR induced recombination. The fragment was cloned into pGem7z (Promega) and sequenced using the M13F primer on an Applied Biosystems 3730×l (Australian Genome Research Facility). Recombination events were identified by sequence analysis.

Controls were carried out to quantify the background rate of recombination produced by the experimental protocol itself. Transfection induced recombination was measured by harvesting plasmid DNA 36 hours post transfection directly from 293T by alkaline lysis as in plasmid DNA preparation from bacterial cells [56]. Plasmid DNA was directly sequenced with NL2944 [AGAGATGGGTGCGAGAG] after isolation by transformation of *E.coli*.

### Molecular clones

Wild-type HIV-1 pNL4-3 plasmid was obtained from the National Institutes of Health AIDS Research and Reference Reagent program, Division of AIDS, NIAID, NIH: pNL4-3 from Dr. Malcolm Martin [57]. Marker HIV-1 pNL4-3 plasmid was created through the introduction of six restriction sites in *gag* by site directed mutagenesis [58], [59], All six sites are codon optimized and have not changed the protein coding sequence, and are separated by 128, 77, 86, 398, 228 base pairs (Figure 2A and 2B). The location of the marker points is determined, in part, by the limited number of locations on the HIV-1 genome where restriction sites can be successfully introduced without changing protein coding sequence.

### Cell culture

293T cells were obtained from the American Type Culture Collection and maintained in DMEM media (Invitrogen) supplemented with 10% vol/vol CCS (Hyclone) and Pen/Strep (Invitrogen). Primary human peripheral lymphocytes (PBLs) were isolated from two independent buffy coats of HIV-1 seronegative blood donors (Red Cross Blood Bank Service, Melbourne) by density gradient centrifugation over Ficol-Plaque Plus (GE Healthcare). PBLs were isolated by counter-current elutriation. The purity of PBLs was assessed by flow cytometry (FACs Calibur; Becton Dickinson) and determined to be >95% pure based on forward scatter and side scatter characteristics. PBLs were stimulated for 2–3 days in RPMI-1640 (Invitrogen) supplemented with 10µg/mL phytohemagglutinin and transferred into fresh RPMI-1640 containing 50 units/mL Interleukin-2 (Roche) before infection.

### Estimating the proportion of heterozygous sequences

We co-transfect equal amounts of WT and MK DNA, in order to produce heterozygous virions. Assuming random co-packaging of viral RNA templates we expect that 50% of the synthesized cDNA to have derived from heterozygous sequences. However, differences in the proportions of the WT and MK sequences may affect the proportion of heterozygous virions (resulting in incorrect estimation of recombination rate). We calculate the expected proportion of heterozygous virions from the experimental data as follows. Let *P_W_* and *P_M_* be the proportion of experimentally observed nucleotide sequence data that is completely WT and MK respectively. *P_W_* and *P_M_* represents cDNA derived from homozygous WT and MK virions, and also cDNA derived from heterozygous virions in which recombination was not observed. Now let *F* be the fraction of cDNA derived from heterozygous virions that did not observe recombination. We then have
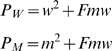
where *w* and *m* are the proportion of WT and MK constructs that were cotransfected into T cells to create the virions. Noting that *m = 1-w* allows for solving the expected proportion of cDNA derived from heterozygous virions, *2mw*. Thus, we do not need to rely on the estimated proportion of WT:MK virus, but can directly estimate it from our data.

### Recombination rate calculation

We measure recombination by infection with two nearly identical HIV-1 viruses, denoted WT and MK, which differ at a number of marker positions in *gag*. Recombination is observed when a single sequence of DNA product contains both WT and MK markers. However, multiple template switches can occur between marker positions, and recombination can only be detected when there are an odd number of template switches. Thus, it is impossible to work out the exact frequency of recombination events. Rather, the data shows the probability of observing recombination (a switch from WT to MK between markers or vice versa) which is calculated as the number of recombination events observed divided by the number of sequences derived from heterozygous infection. Denote the probability of observing a recombination event between two marker positions separated by a genomic distance of *L* as *R(L)*. Denote the recombination rate per nucleotide per round of infection as *r*. These two quantities then satisfy the following.
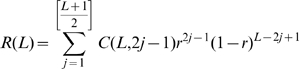
where *[(L+1)/2]* is the integer part of *(L+1)/2* and *C(L,i)* is the binomial coefficient for picking i unordered outcomes from *L* possibilities. Alternatively, when the genomic distance *L* is sufficiently large and the recombination rate *r* is sufficiently small (as is generally the case with recombination experiments) the following Poisson approximation holds [36] (see ‘Poisson approximation’)
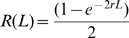
which can be re-arranged to calculate the recombination rate as
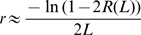
(A)Finally if recombination is studied over multiple regions of lengths *L_1_*, *L_2_,L_3_,…,L_k_*, then the recombination rate, *r*, is calculated as the *r* value that minimises the chi-square value
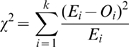
(B)Where *O_i_* and *E_i_* is the observed and expected number of template switches that is detected in region *i* respectively. The expected number of template switches is calculated as the multiple of *R(L_i_)* and the number of heterozygous sequences.

### Comparing recombination rates

Probability distributions were generated by bootstrapping the sequence data as follows. In each bootstrap loop, sequence data was randomly sampled with replacement until the same number of sequences that were originally sampled, had been sampled *in silico*. From each new sample set the optimal rate of recombination was calculated as described above. This bootstrapping procedure was completed 10000 times and pooling each bootstrap loop generates a probability distribution for the recombination rate, *r*. Note that we sampled from the entire sequence pool, and thus this approach also incorporates the level of uncertainty in the proportion of heterozygous virus in the sample. The probability distributions of the recombination rate for different genetic constructs/target cells was used to compare rates.

### The ratio of GFP+ and infection MOIs represent the probability of an infection event resulting in the reconstruction of a functional GFP protein

Let *s(n)* be the probability that a single cell has been infected *n* times with the HIV reporter virus. Let *m* be the MOI for the HIV reporter virus. Let *p_GFP_* be the probability of a single infection event resulting in the reconstruction of a functional GFP encoding region. That is, the probability that an infecting virion is heterozygous, and that recombination occurred between the two co-packaged RNAs such that both GFP deactivating mutations are eliminated. Then, the probability that a single cell has *n* infections that reconstitute a function GFP is given by
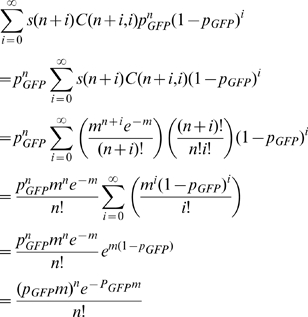
which is the MOI formula (Poisson distribution) with MOI equal to the product, *p_GFP_m*. Note that *C(n+i,i)* is the binomial coefficient for picking *i* unordered outcomes from *n+i* possibilities. Thus, the GFP MOI equals *p_GFP_m* and division of the infection MOI, *m*, leaves *p_GFP_*, the probability that an infection event will reconstitute a functional GFP encoding region.

### Control correction

In this assay, recombination can occur at two independent stages: The experimentally induced recombination, and the viral reverse transcription induced recombination. We measure the experimentally induced recombination alone, and the cumulative effect of experimentally induced recombination with the reverse transcription induced recombination. From this we calculate the reverse transcription induced recombination rate alone as follows. Let R_E_(L) and R_R_(L) be the probability of observing recombination over a genomic distance of L for the experimentally induced and RT induced recombination rates respectively. The cumulative probability of observing recombination after both effects, *R(L)*, is given by
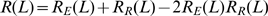
Note that R_E_R_R_ is subtracted once as R_E_ and R_R_ are independent and not mutually exclusive events, and subtracted a second time to eliminate the cases where PCR template switch nullifies an RT template switch. This is then re-arranged to give
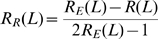
The recombination rate is calculated from equation (A). If recombination is measured over multiple regions, as is the case in our experimental system, this should be applied to each region before calculating the recombination rate by minimizing the chi-square value (equation B).

### Poisson approximation

The binomial terms *P_i_(L)* above can be approximated by the Poisson distribution when the length, *L*, is sufficiently large, and the recombination rate, *r*, is sufficiently small. Under these conditions the Poisson coefficient is the product of the genomic length and recombination rate *Lr*. The probability of observing recombination is then approximated by
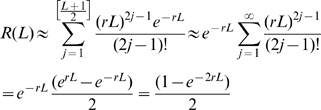
Thus, to calculate the recombination rate *r* from experimental data we re-arrange to give
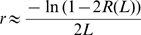
where *R(L)* can be measured from experimental data as the proportion of heterozygous sequences over which recombination was observed.

## Supporting Information

Figure S1 (A) Replication kinetics of WT and MK virus. Equivalent levels of virus, as determined by a micro-RT assay, were added to 2×10^5^ PHA stimulated PBMCs in triplicate. Seven 10-fold serial dilutions of each virus, and a no virus control were tested in triplicate. Supernatants were collected on days 3, 7, 10 and 14 post-infection and viral production was measured using a micro-RT assay. (B) Protein processing profiles of cellular and virion lysates. 293T cells were transfected with WT and MK plasmid. 36 hours post-transfection, cells were washed twice in DPBS and pelleted at 1,462×g for 10 min at 4°C. Viral particles were purified and concentrated by ultracentrifugation through a 20% sucrose cushion using a Beckman ultracentrifuge L-90 model (SW 41 rotor) at 100,000×g for 1 h at 4°C. Cell and virion pellets were lysed in TBS lysis buffer (50 mM Tris-HCl [pH7.4], 150 mM NaCl, 1% vol/vol NP-40, 20 mM phenylmethylsulfonyl fluoride (PMSF), 1 µM pepstatin, and 1 µM leupeptin) at a concentration of approx 1×10^7^ cells or 40 µg of p24 per mL. Cell lysates were rapidly freeze-thawed three times to weaken the cellular membrane and cell debris was subsequently removed by centrifugation at 20,000×g for 30 min at 4°C. Lysates were mixed with 5× loading buffer (100 mM Tris-HCl [pH 6.8], 1.6% β-mercaptoethanol, 3% SDS, 33% glycerol and 0.3% bromophenol blue), incubated at 95°C for 5 min and resolved by SDS polyacrylamide gel electrophoresis (SDS-PAGE). Resolved proteins were transferred to a nitrocellulose membrane (Amersham). The membrane was incubated for 30 min in blocking buffer (5% wt/vol skim milk, 50 mM Tris-HCl [pH7.4], 150 mM NaCl) at room temperature. Proteins were identified using pooled HIV-1 seropositive patient sera.
(1.52 MB TIF)
