# Supplementary figures and images for: Accurately Measuring Recombination between Closely Related HIV-1 Genomes
Source: PLoS Comput Biol. 2010 Apr 29;6(4):e1000766. doi: 10.1371/journal.pcbi.1000766 (PMC2861704; doi:10.1371/journal.pcbi.1000766)

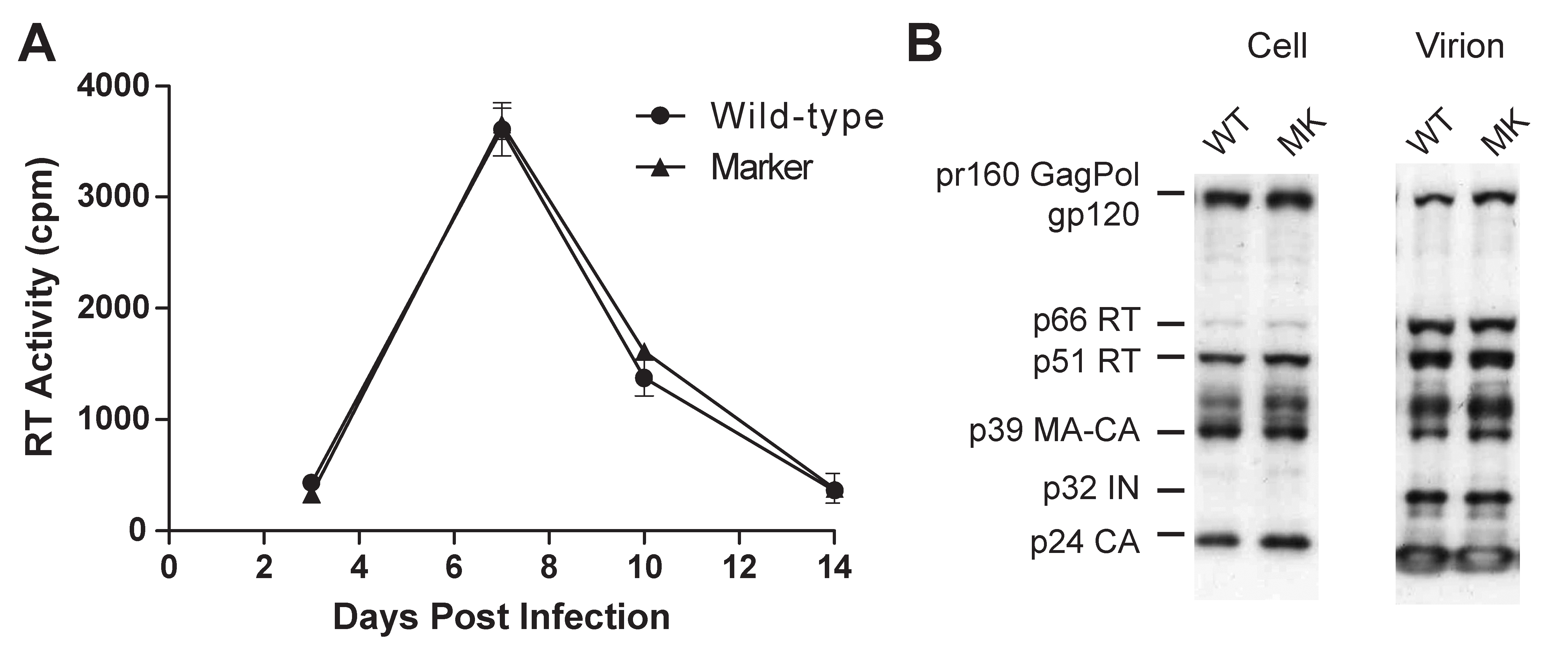

Supplement: Figure S1 — (A) Replication kinetics of WT and MK virus. Equivalent levels of virus, as determined by a micro-RT assay, were added to 2×10^5^ PHA stimulated PBMCs in triplicate. Seven 10-fold serial dilutions of each virus, and a no virus control were tested in triplicate. Supernatants were collected on days 3, 7, 10 and 14 post-infection and viral production was measured using a micro-RT assay. (B) Protein processing profiles of cellular and virion lysates. 293T cells were transfected with WT and MK plasmid. 36 hours post-transfection, cells were washed twice in DPBS and pelleted at 1,462×g for 10 min at 4°C. Viral particles were purified and concentrated by ultracentrifugation through a 20% sucrose cushion using a Beckman ultracentrifuge L-90 model (SW 41 rotor) at 100,000×g for 1 h at 4°C. Cell and virion pellets were lysed in TBS lysis buffer (50 mM Tris-HCl [pH7.4], 150 mM NaCl, 1% vol/vol NP-40, 20 mM phenylmethylsulfonyl fluoride (PMSF), 1 µM pepstatin, and 1 µM leupeptin) at a concentration of approx 1×107 cells or 40 µg of p24 per mL. Cell lysates were rapidly freeze-thawed three times to weaken the cellular membrane and cell debris was subsequently removed by centrifugation at 20,000×g for 30 min at 4°C. Lysates were mixed with 5× loading buffer (100 mM Tris-HCl [pH 6.8], 1.6% β-mercaptoethanol, 3% SDS, 33% glycerol and 0.3% bromophenol blue), incubated at 95°C for 5 min and resolved by SDS polyacrylamide gel electrophoresis (SDS-PAGE). Resolved proteins were transferred to a nitrocellulose membrane (Amersham). The membrane was incubated for 30 min in blocking buffer (5% wt/vol skim milk, 50 mM Tris-HCl [pH7.4], 150 mM NaCl) at room temperature. Proteins were identified using pooled HIV-1 seropositive patient sera. (1.52 MB TIF) [file pcbi.1000766.s001.tif]
